# Apoptotic Mechanisms of Quercetin in Liver Cancer: Recent Trends and Advancements

**DOI:** 10.3390/pharmaceutics15020712

**Published:** 2023-02-20

**Authors:** Gautam Sethi, Prangya Rath, Abhishek Chauhan, Anuj Ranjan, Renuka Choudhary, Seema Ramniwas, Katrin Sak, Diwakar Aggarwal, Isha Rani, Hardeep Singh Tuli

**Affiliations:** 1 Department of Pharmacology, Yong Loo Lin School of Medicine, National University of Singapore, Singapore 117600, Singapore; 2 Amity Institute of Environmental Sciences, Amity University, Noida 201303, India; 3 Amity Institute of Environmental Toxicology, Safety and Management, Amity University, Noida 201303, India; 4 Academy of Biology and Biotechnology, Southern Federal University, 344090 Rostov-on-Don, Russia; 5 Department of Biotechnology, Maharishi Markandeshwar Engineering College, Maharishi Markandeshwar (Deemed to be University), Mullana, Ambala 133207, India; 6 University Centre for Research and Development, University Institute of Pharmaceutical Sciences, Chandigarh University, Gharuan, Mohali 140413, India; 7 NGO Praeventio, 50407 Tartu, Estonia; 8 Department of Biochemistry, Maharishi Markandeshwar College of Medical Sciences and Research (MMCMSR), Sadopur, Ambala 134007, India

**Keywords:** Quercetin, liver cancer, apoptosis, caspases, JAK-STAT, autophagy, cell signaling

## Abstract

Due to rising incidence rates of liver cancer and worries about the toxicity of current chemotherapeutic medicines, the hunt for further alternative methods to treat this malignancy has escalated. Compared to chemotherapy, quercetin, a flavonoid, is relatively less harmful to normal cells and is regarded as an excellent free-radical scavenger. Apoptotic cell death of cancer cells caused by quercetin has been demonstrated by many prior studies. It is present in many fruits, vegetables, and herbs. Quercetin targets apoptosis, by upregulating Bax, caspase-3, and p21 while downregulating Akt, PLK-1, cyclin-B1, cyclin-A, CDC-2, CDK-2, and Bcl-2. Additionally, it has been reported to increase STAT3 protein degradation in liver cancer cells while decreasing STAT3 activation. Quercetin has a potential future in chemoprevention, based on substantial research on its anticancer effects. The current review discusses quercetin’s mechanisms of action, nanodelivery strategies, and other potential cellular effects in liver cancer.

## 1. Introduction

Globally, liver cancer is the third most frequent cause of mortality despite a significant amount of research related to its treatment. It is more common in men than in women. Scientists from IARC published and submitted a report to WHO, which stated the worldwide diagnosis of people with liver cancer in 2020 was 905,700, out of which 830,200 people died from this disease. Global age-standardized incidence and mortality rates (ASRs) were also reported in their study, which reported an ASR of 9.5 for new cases and 8.7 for deaths in liver cancer per 100,000 people. Based on the most recent estimates of the worldwide burden of liver cancer in 2020, it was predicted that by 2040, the number of cases and fatalities from liver cancer would increase by more than 55% [1].

The treatment of cancer includes various traditional methodologies such as radiotherapy, chemotherapy, surgery, immunotherapy alone or in combination [2,3,4]. However, efficacy of the methods was greatly reduced by their limitations, such as sensitivity of normal cells to irradiation [5], chemotherapeutic drug resistance [6,7,8], poor liver functional reserve, incomplete tumor resection [9] and development of intrinsic or acquired resistance [10,11,12]. To overcome the disadvantages of the present methodologies, the discovery of novel anticancer agents with improved efficacy and minimal side effects continues.

Phytotherapy is one of the potential options involving the usage of plants for the production of traditional drugs in the treatment of various cancers [13,14,15]. Nowadays, application and evaluation of anticancer therapeutic effects of plants and their compounds is increasing. However, the mechanism by which these drugs act as anticancer agents is mostly unclear. Though the induction of antioxidant effects in the prevention and treatment of cancer is obvious, as plants are good sources of antioxidants [16,17,18]. A study has shown the antioxidative properties present in soil algae (*Pleurochloris pyrenoidosa*, *Botrydiopsis eriensis*, and *Scenedesmus obliquus*) which was attributed to the presence of flavonoid compounds like quercetin [19]. In addition, another report has described anticancer and antioxidant activities of some algae such as Chlorophyta (*Ulva lactuca*, and *Codium tomentosum*), Phaeophyta (*Cystoseira crinita*, *Cystoseira stricta*, and *Sargassum vulgare*), and Rhodophyta (*Gelidium latifolium*, *Hypnea musciformis*, and *Jania rubens*). These algae comprised sources of polyphenols, such as flavonoids, isoflavones, cinnamic acid, benzoic acid, quercetins, etc. [20]. Most studies have suggested that the prevalence of cancer is lower in people consuming more fruits and vegetables that have antioxidative effects. In different plants having biological activities, there are more than 25,000 phytochemicals. From 1940 to 2014, more than 50% of the approved anticancer drugs originated from natural sources [21].

Quercetin is a flavonoid that exists in daily dietary foods such as apple, red grapes, broccoli, onion, citrus and black-berry. Studies have reported that it may play a crucial role in the prevention or treatment of various diseases such as cancer. Therefore, it has been added to functional foods as a commercial dietary supplement [22]. Over the past years, numerous studies have reported its biological functions, such as anti-inflammatory, anti-oxidant and anti-cancer effects [23]. Quercetin possesses the capability to regulate mechanisms such as inflammation, fibrosis, migration, apoptosis, and angiogenesis, involved in the progression of hepatocellular carcinoma [24]. Additionally, it inhibits the inflammatory enzymes and also modulates oxidative stress via ROS depletion, which further enhances the antioxidant system [25,26]. In hepatocellular carcinoma, quercetin has been shown to have antiproliferative and anticancer effects through inducing cell cycle arrest, inhibiting the production of cyclins, inducing CDK inhibitors, inhibiting metabolic activity, inducing cell death, and inhibiting survival signals [27,28,29,30,31,32]. Recent studies have also reported that quercetin has the capability to reduce tumor microenvironment components and can be used for hepatocellular carcinoma growth inhibition [33]. Until now, very limited mechanistic information is available describing the role of quercetin in liver cancer prevention with improved efficacy, in contrast to traditional methods. Therefore, in this review, we provide a thorough and contemporary overview about the recent and eminent research on the role of quercetin in liver cancer, especially through regulating apoptotic mechanisms.

## 2. Chemistry of Quercetin

**Figure 1:**
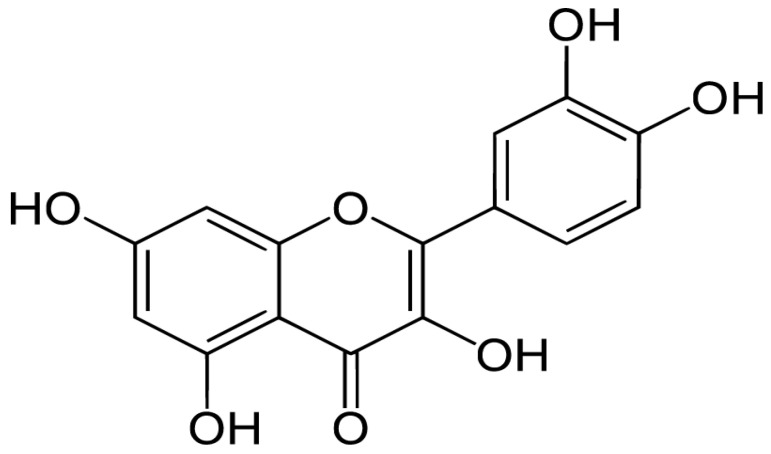
Chemical structure of quercetin.

Quercetin, also known as 3′,4′,5,7-tetrahydroxyflavonol, belongs to the flavonols (flavonoids) and is mostly found in fruits, black and green tea, beans, and vegetables as a secondary metabolite [34]. It is present in conjugated forms with sugar moieties [35]. It possesses five -OH groups placed at the 3-, 3′-, 4′-, 5- and 7-positions. X-ray crystallography of quercetin gave crystal data as follows: a = 13.060(5), b = 16.564(7), c = 3.725(2) Å, α = 92.05(4), β = 94.39(3), γ = 120.55(3), V = 689.4(5) Å3, z = 2, space group P1, Dc = 1.63 g cm^−3^, Dm = 1.69(1) g cm^−3^ [36]. In crystal form, it exists as H-bonded dimers. These dimers form a 2-D net connected through water molecules. It can exist in a free state in the form of aglycone, or as its derivative and conjugates with carbohydrates, known as quercetin glycosides; with alkyls, known as quercetin methyl or ethyl; with hydroxyl groups, known as quercetin ethers; and with sulfate groups, known as quercetin-derived sulfates. Two phenyl groups are connected by three carbon bridges to form the basic structure of flavonoids. A diversity of flavonoid structures result from variations in ring-C and substitution patterns in rings-A and -B. Due to the substitutions of various functional groups on the main flavonol molecule, a broad range of biochemical and pharmacological properties are observed [37]. Three structural groups contribute to quercetin’s stability. These operate as an antioxidant and confer antioxidative properties by countering free electron carrying species, such as the B ring o-dihydroxyl groups, the 4-oxo group conjugated to the 2,3-alkene, and the 3- and 5-hydroxyl groups [34]. The structure of quercetin is provided in Figure 1.

## 3. Absorption and Metabolism

Flavonoids are poorly absorbed. A small quantity of ingested quercetin gets absorbed in the stomach; however, the small intestine is found to be the major absorption site. They enter the colon and are digested by enterobacteria into an aglycone. Due to its lipophilicity, it is then quickly absorbed in the large intestine and is then metabolized in the liver by O-methylation, glucuronidation, and/or sulfation. [34]. Following intestinal absorption, it proceeds through phase-II metabolism before being ultimately eliminated by the liver into bile or via the kidneys into urine. Quercetin and its glycosides are mostly transported by sodium-dependent glucose co-transporters (SGLTs), which are found on the apical membrane of intestinal epithelial cells [38]. Additionally, quercetin modifies the intestinal microbiota and protects the intestinal barrier [39]. Quercetin glucosides can pass through epithelial cell layers and get transported into the circulatory system. They are less effective than quercetin aglycone, though. As a result, it has been discovered that quercetin absorption is accelerated by the hydrolysis of the glucoside to the aglycone [40]. According to research, the type and positioning of the sugar moieties connected, determine how well quercetin is absorbed [34]. Computational studies to analyze the absorption, distribution, metabolism and excretion have also been carried out [37]. A study has revealed that the plasma binding protein ability rate of quercetin and its derivatives were within the range of 85.36–99.82. This study also showed prediction of ADME properties of quercetin and its derivatives using the ACD/I-Lab platform which revealed that quercetin and the 3′-methyl ether quercetin were found to have maximum passive absorptions of 100%, while other quercetin derivatives showed less than 15% absorption [37]. The intestine and liver are the main sites for quercetin metabolism. Quercetin passively diffuses through OATP2B1, OATP1A2, and OCT1 in HEK293 cell lines. A study revealed that in humans, quercetin gets absorbed in the small intestine where it is catabolized by the gut microbiota-derived β-glucosidase and lactasephlorizin hydrolase and the products are absorbed by colon. Quercetin monoglycosides get absorbed by SGLT-1 [41]. The resulting compounds circulate in blood as conjugates with attached glucuronide, methyl, or sulfate groups [42]. These metabolites are transported through MRP-2 and subsequently to the liver through blood vessels. They are then exposed to biotransformation enzymes and undergo secondary metabolisms I and II [43]. In phase I metabolism it undergoes oxidation, reduction, and hydrolysis, which increases the reactivity and facilitates its subsequent metabolism. The resultant products undergo phase II metabolism including glucuronidation, sulfation, and methylation reactions, which benefits its excretion through bile and urine [39]. Quercetin and its metabolites are capable of passing the blood–brain barrier. Quercetin-3-O-β-D-glucuronide is the main metabolite of quercetin that gets transported to target tissues through plasma and exerts its biological activity in the targeted tissues. The half-life of quercetin metabolites varies from 11–28 h [44].

## 4. Apoptotic Mechanisms of Quercetin

### 4.1. Activating Caspase Proteases

**Figure 2:**
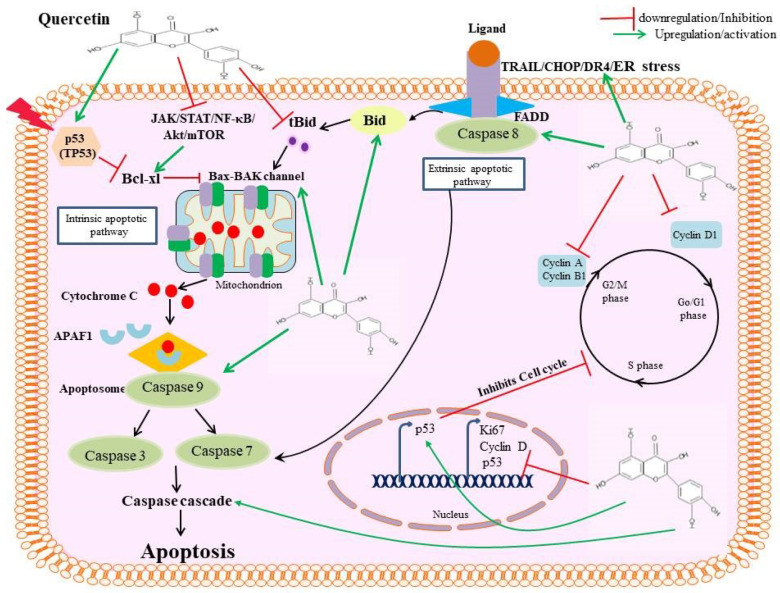
Pictorial representation showing anti-apoptotic effect of quercetin including both extrinsic and intrinsic apoptotic pathways in cancer cells.

The actions of the caspases (family of cysteine proteases) are intimately related to the apoptotic cell death process. Caspases are first created as monomeric, inactive procaspases that must dimerize, and frequently cleave, in order to become active [45]. Caspase activation is a terminal event in the apoptotic process, not a direct activation specifically caused by quercetin. Therefore, it is essential to understand that caspase activation is a molecular mechanism involved in quercetin-induced apoptosis. By activating the caspases in the human hepatoma cell line HepG2, quercetin causes apoptosis. Treatment of the cells for 18 h induced apoptosis by activating caspase-3 and -9 [30]. Similarly in J/Neo cell lines, quercetin-induced apoptosis was found to activate caspase-9 and caspase-3 in a dose-dependent manner [46]. Quercetin has been observed to induce proteolysis of vimentin by activating caspase-3. This results in a decrease in the cancer stem cell population present in a human papillary thyroid cancer cell line [47]. Researchers have examined the effects of compounds derived from quercetin on several types of HCC cell lines and have noted their pro-apoptotic and anti-proliferative capabilities, which are related to caspase activity [24]. Quercetin administration at 60 mg/kg orally to mice with SMMC-7221 HCC cells was responsible for cleaving caspase-3 protein levels [48]. Reduced cleaved caspases-9 and -3 were seen after quercetin encapsulation (PLGA-loaded gold–quercetin nanoparticles) was administered to MHCC97H xenograft mice models at doses of 30, 40, and 50 mg/kg [49]. Researchers revealed that 10 mg/kg of co-encapsulated quercetin and sorafenib enhanced the expression of the caspase-3 protein [50]. In SMMC7721 cells, quercetin at 20 µM leads to cleavage of caspase-3, and procaspase-3 into a p20 intermediate, thereby leading to liver cancer cell apoptosis [51]. A study has shown that it affects enzymatic activity of caspase-8 after 4–18 h of incubation in all quercetin concentrations. With 25 mol/L quercetin, HepG2 cells were treated for 18 h, which enhanced both the levels of active and cleaved caspase-3 [30]. Similarly, another study has shown that caspase-9 was cleaved by quercetin polyphenol in a rat hepatoma cell line H4IIE [52]. It has been shown that procaspase-3 levels were regulated by quercetin TRAIL-resistant hepatocellular carcinoma cells [53]. Quercetin has also been reported to suppress caspase-3 expression, elevate p53 expression, inhibit cell proliferation, downregulate cell cycle markers cyclin D1 and Ki-67 [29]. Figure 2 represents various apoptotic mechanisms mediated by quercetin.

### 4.2. Modulating the Bcl2-Bax Pathway

In addition to taking part in caspase-dependent apoptosis, the mitochondria also have a major impact on the Bcl-2 pathway during caspase-independent apoptosis. At least one of the four homologous areas known as Bcl homology (BH) domains (BH1 to BH4), regulate Bcl2 protein interactions [54]. The Bcl-2 family substrates have been seen to become most activated in response to quercetin. It assisted in Bcl-2 regulation of HepG2 and boosted translocation of Bax to the mitochondrial membrane while lowering the Bcl-xL:Bcl-xS ratio [30]. Another study found that quercetin-treated J/Neo and J/Bcl-xL cells exhibited downregulation of SQSTM1/p62 protein levels as well as autophagic events such as the Akt-mTOR pathway, formation of acidic vesicular organelles, conversion of microtubule-associated proteins such as light chain 3-I (LC3-I) to LC3-II, and formation of acidic vesicular organelles [46]. When quercetin and an autophagy inhibitor like chloroquine are combined, Bak activation, which triggers the mitochondrial damage-mediated apoptosis pathway, is significantly increased [46]. Quercetin has been shown to enhance the fraction of cells in the G0/G1 phase and to regulate Survivin and Bcl-2 in HepG2 cell death [55]. Quercetin administration at 60 mg/kg orally to mice with SMMC-7221 HCC cells have increased Bax protein levels and decreased Bcl-2 protein expression [48]. Quercetin also potentiated doxorubicin mediated anticancer effects in liver cancer cells by regulating p53/Bcl-xl pathways [51]. When quercetin (50 μmol/L) was administered, the Bcl-xL:Bcl-xS ratio fell and eventually reached a minimal value [30]. Quercetin controls the expression of Bcl-xL, Bcl-xS, and Bax in several ways. This provided evidence that the control of the apoptotic process may depend on the balance of expression of these proteins [52]. Quercetin treatment of HCC cells significantly upregulated the mRNA and protein levels of death receptor TRAIL, transcription factor Sp1, and expression of Bcl-xL [53]. Interaction between proteins playing a role in regulating cell death such as Bad, Bcl-xL, Bak, etc. were regulated in many cancer cell lines by quercetin administration [29]. Lower levels of anti-apoptotic Bcl-xL and higher levels of proapoptotic Bcl-2 family members including Bcl-xS and Bax have been demonstrated to directly contribute to the cell apoptotic process. According to a recent study, quercetin inhibits the development of liver fibrosis via regulating the activity of the NF-κB/IκB, p38 MAPK, and Bcl-2/Bax signaling pathways in hepatic stellate cells (HSCs) [56].

### 4.3. Targeting the PI3K-Akt-mTOR Pathway

One of the most often over-activated intracellular pathways in a number of human malignancies is the PI3K/AKT pathway. This pathway leads to the development of cancer, tumor cell proliferation, invasion, and metastasis by acting on many downstream target proteins [57]. In a human hepatoma cell line, quercetin caused the PI-3-kinase/Akt and ERK pathways to be inhibited (HepG2). In cells treated with quercetin, a long-lasting suppression of Akt and extracellular regulated kinase (ERK) also took place [30]. Exposure of quercetin in SMMC-7721, BEL-7402 HCC cells has shown altered Akt/mTOR inhibition by decreasing p-Akt/Akt and p-mTOR/mTOR rates [58]. It decreased the protein levels of HK2 and thereby suppressed the AKT/mTOR pathway in HCC cells [58]. Quercetin treatment of SMMC-7721 and HepG2 HCC cells have shown decreased p-Akt, p-mTOR, p-p70S6K and p-4EBP1 protein levels thereby targeting Akt/mTOR inhibition and MAPK activation [48]. Administration of encapsulated quercetin at a dose of 30, 40 and 50 mg/kg to MHCC97H xenograft mouse models has shown decreased p-Akt, p-ERK1/2 protein, leading to Akt/ERK1/2 inhibition [49]. Induction of p53 as a consequence of PI3K and PKC downregulation has been associated with chemo preventive effects in liver cancer cells BEL-7402 HCC [59]. Quercetin was observed to inhibit inflammation in liver through NF-κB/TLR/NLRP3, and also reduced PI3K/Nrf2 mediated oxidative stress, reduced mTOR activation, and also inhibited the expression of apoptotic factors/proteins associated with liver disorders and cancers [60]. Quercetin also led to a significant increase in autophagosomes and autophagolysosomes in hepatocellular carcinoma (HCC) cells. It was observed that quercetin also stimulated autophagy by inactivating the AKT/mTOR pathway and activating the MAPK pathway [61]. By blocking the MEK1/ERK1/2 signaling pathway and subsequently reducing the proteasome’s subunits in HepG2 cancer cells, it reduced the chymotrypsin activity of the proteasome [62]. Quercetin concentrations more than 50 mol/L were found to inhibit Akt via lowering the amount of phosphorylated active Akt [30].

### 4.4. Targeting JAK-STAT3 Signal Pathway

Signals from cytokines, interleukins, and growth factors are sent through a number of transmembrane receptor families in JAK/STAT pathways. Research revealed that a successful drug development technique has been to target these intracellular signaling networks [63]. Treatment of LM3 cells with quercetin decreased p-STAT3 protein expression, and targeted JAK2/STAT3 inhibition [32]. Administration of quercetin led to suppression of liver tumors by targeting cell proliferation via activation of the JAK/STAT signaling route. Treatment with quercetin regulated the effect of signal transducer and activator of transcription-1 (STAT1) tyrosine phosphorylation, and elevated IFN-β-induced STAT1 tyrosine phosphorylation in HepG2 cells, thereby activating the JAK/STAT pathway [64]. Quercetin treatment also inhibited M1 macrophage polarization after injury through inhibiting STAT1 and NF-κB pathways [65]. Studies using immunocytochemistry were performed to evaluate the nuclear STAT3 levels. The study results revealed that quercetin successfully inhibited the proliferation of liver cancer cells in a dose- and time-dependent manner. It also led to an increase in sub-G0/G1 apoptotic populations [66]. It reduced the expression of p-JAK1 and p-STAT3 while it decreased STAT3-dependent activity in many hepato-cancerous cells [66]. Treatment of cancer cells with 25, 50, and 100 µM doses of quercetin, led to dose-dependent apoptosis of cell lines, which resulted in decreased STAT3 phosphorylation levels [67]. Similar effects were observed in Huh7 cells where STAT1 levels were regulated by quercetin treatment [64,67]. It potentiated the inhibitory effect of IFN-α on hepato-cancer cell proliferation by activating JAK/STAT pathway signaling through inhibiting SHP2 [64]. It also played a suppressive role against HCC cells by initiating apoptosis and p16-mediated cell cycle arrest, thereby suppressing cancer cell growth [68].

### 4.5. Inducing Apoptosis via Autophagy Modulation

**Figure 3:**
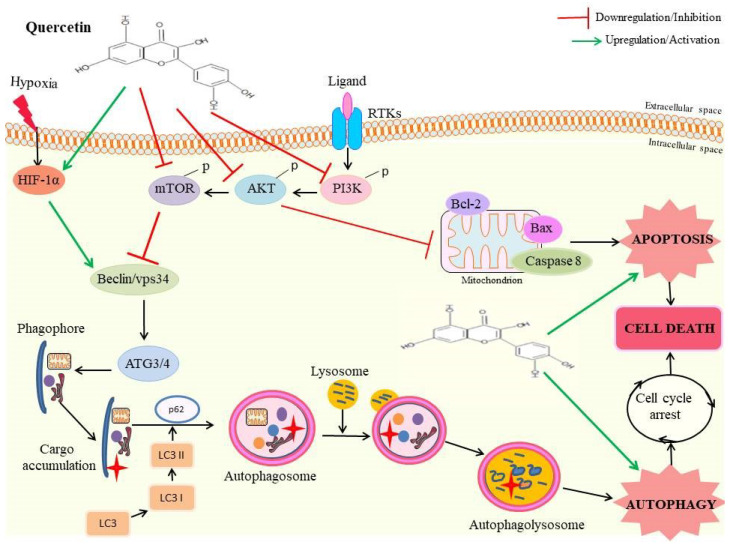
A representation of involvement of quercetin in both autophagic and apoptotic regulation through various signaling mechanisms.

Under starvation conditions, autophagy induction is typically thought to act as a cancer defense mechanism. However, prolonged starvation stress causes the tumor tissues to consume themselves. Autophagy performs this role, making it simpler for oncogenic substances to be broken down and so slowing the growth of tumors, in contrast to apoptosis, which kills cancer cells via programmed cell death [69]. Quercetin has been observed to modulate apoptotic and autophagic cell death pathways in many cancerous cells (Figure 3). It limits initiation, differentiation, and proliferation of cancerous cells. Quercetin has been observed to inhibit growth of hepatocellular carcinoma cells by inducing apoptosis through autophagy stimulation in mouse models. It has been observed to increase auto-phagosome fusion with lysosomes and forms auto-lysosomes in HCC cell lines. These inhibit the Akt/mTOR pathway, thereby activating the MAPK pathways [48]. Treatment with quercetin has been observed to induce formation of intracellular autophagic vacuoles that later form auto-phagosome/auto-lysosomes. This then leads to cell cycle arrest and onset of apoptotic cell death. Quercetin treatment reduced phosphorylation of proteins such as p70S6 and 4E-BP1 [70,71]. A study has shown that quercetin induced protective autophagy in gastric cancer cell lines. This was attributed to the involvement of Akt-mTOR as well as HIF-1α mediated signaling. It also led to formation of acidic vesicular organelles, conversion of LC3-I to LC3-II, recruitment of LC3-II to auto-phagosomes and activation of autophagy genes [72].

## 5. Synergism of Quercetin in the Liver Cancer

Tumors can easily develop resistance to a single oncolytic drug since they are composed of genetically diverse clones. Consequently, synergistic targeted therapy is the best course of action for cancer [73,74,75,76]. Combining two or more drugs, each with a unique anti-tumor mechanism, results in synergism, which further strengthens the anti-tumor action without affecting the normal, healthy cells. Numerous targeted therapies have been discovered for the treatment of liver cancer, but none have shown much efficacy against cancer cells. Quercetin showed synergistic effects when used with other anti-cancer compounds. Studies have reported the synergistic effect of quercetin when used with 5-FU in liver cancer cell lines. This combination led to enhanced growth inhibition in some cell lines, in comparison to quercetin administration alone [28,77]. Another study discovered that quercetin alone or in conjunction with sorafenib, the first drug approved to treat advanced hepatocellular carcinoma, all downregulated the anti-inflammatory, proliferative, and angiogenesis-related genes TNF-, VEGF, P53, and NF-B. HCC growth was significantly inhibited by treatment with sorafenib and quercetin, which also produced cell cycle arrest, apoptosis, and necrosis [78]. Quercetin can boost ZD55-TRAIL mediated growth inhibition and death in HCC cells, according to research on the synergistic anti-tumor effects of quercetin and oncolytic adenovirus in HCC. Quercetin combination has shown promise in both in vivo and in vitro anti-HCC trials [79]. In drug-resistant cancers, particularly liver cancer, the clinical applications of doxorubicin (DOX) are limited due to dose-dependent toxicities. The study on the combined use of DOX and quercetin indicated an enhanced anti-tumor activity in liver cancer cells through p53/Bcl-xl, and protection of the normal liver cells [51]. In a different study, quercetin’s synergistic effect with cisplatin, a common chemotherapeutic agent, was examined using human hepatocellular carcinoma cells. It was found that quercetin had suppressive effects through p16-mediated cell cycle arrest and death. The inhibitory effects in suppressing cell growth and inducing apoptosis were more when used in combination [68]. Because of this, the development of quercetin may be advantageous in a combination therapy that inhibits the growth of liver cancer cells more severely while sparing healthy cells. The respective combinations can increase the therapeutic efficacy against liver cancer.

## 6. Nano Delivery of Quercetin in Liver Cancer

Due to quercetin’s poor water solubility and delivery, low bioavailability, chemical instability, and brief half-life, its clinical use in cancer chemoprevention is constrained. Quercetin accumulation and bioavailability in the liver can be enhanced by controlled drug delivery methods such nano conjugation [80,81]. Nano conjugated quercetin has garnered a lot of interest due to its prospective therapeutic applications, regulated drug release, prolonged retention in tumors, and increased anticancer potential. Liposomes, silver nanoparticles, silica nanoparticles, poly (D,L-lactic acid), poly (lactic-co-glycolic acid), polymeric micelles, chitosan nanoparticles, and other drug carriers are used to deliver effective outcomes [82]. The flavonoid nano formulations’ anti-cancer activity may be explained by a number of mechanisms, including activation of caspase enzymes, induction of cell cycle arrest, reduction in tumor vascularization, reduction in tumor cell invasion and metastasis, induction of mitochondrial damage, and apoptosis [83]. According to reports, the use of quercetin-PLG with polymeric nanoparticles in the treatment of liver cancer resulted in effects such as reduced release of cytochrome C from mitochondria, cytosolic SOD, increased glutathione-one-D-transferase, and inhibition of lipid peroxidation leading to cell cycle arrest. Use of liposomal nanoparticles with quercetin have been reported in liver cancer and showed its effect by the downregulation of HSP70 and cell cycle arrest. Gold particles with a size of 106.7 nm with quercetin, have also been reported in liver cancer and the effects included release of cytochrome c through cleavage of caspase-3 and caspase-9, decrease in COX-2 via suppression of NF-κB nuclear translocation and its binding to the COX-2 promoter and the inactivation of Akt and ERK1/2 signaling pathways [84]. Table 1 summarizes delivery systems for quercetin developed against different cancers.

## 7. Safety Aspects

In general, quercetin is considered to be safe. This statement has also been verified in several human intervention studies, reporting only rarely some mild adverse effects following the intake of quercetin supplements [91]. For instance, no severe adverse events were detected among chronic obstructive pulmonary disease patients after administration of quercetin up to 2000 mg/day for one week [92]. Similarly, quercetin displayed safety among patients suffering from chronic hepatitis C virus infection, even at doses as high has 5 g per day for 4 weeks [93]. However, possible interactions of quercetin with other drugs cannot be excluded and should be analyzed case by case. Table 2 and Table 3 represent summaries of diverse preclinical investigations carried out using quercetin in liver cancer.

## 8. Conclusions

As shown in this review article, quercetin may play several important roles in the fight against liver cancer, inducing cell cycle arrest and apoptotic cell death through modulating various intracellular mechanisms. Therefore, this plant secondary metabolite could be considered a novel potential anticancer drug candidate. However, many issues must be solved first before initiating clinical trials with liver cancer patients. Firstly, the most appropriate formulation of quercetin should be developed to avoid its low bioavailability and extensive metabolic conversion in the human body. Secondly, the proper combination with the current cytotoxic and/or targeted drugs must be elaborated, allowing reduction in the doses of conventional therapeutics and thereby also their toxicities. Nevertheless, the data compiled in this review article clearly highlight the importance of quercetin in the future management of malignant neoplasms in the liver.

## Figures and Tables

**Table 1:** Anti-Cancer effects of various types of quercetin nano-formulations based delivery systems.

S No.	Type of Nano Formulations/ Nanoparticles (Quercetin Based)	Type of Cancer	Study Model (Both In Vitro/In Vivo)	Particle Size (Nanometres (nm))	Effects	Concentration	References
1	PLGA nanoparticles	Cervical	HeLa	89.8 nm	↑ apoptosis ↑ Caspase-3 and 7	1, 10, 25 and 50 µM	[85]
2	PLGA nanoparticles	Breast	MCF-7	89.8 nm	↑ apoptosis frequency, ↑ Mitochondrial damage in Cancer Cells,	1, 10, 25 and 50 µM	[85]
3	PLGA nanoparticles	Breast	DMBA-induced mammary adenocarcinoma SD rats	89.8 nm	↓ average number of tumors and prolonged the tumor latency period	128 mg/kg	[85]
4	Poly (lactic-co-glycolic acid) nanoparticles	Glioma	C6	Qu1NP-215.2 nm, Qu2NP-282.3, Qu3NPs-584.5 nm	↓ cancer cell proliferation, ↑ SOD activity, ↑ GSH levels	1–100 μg/mL	[86]
5	Phenylboronic acid (PBA) conjugated Zinc oxide nanoparticles (PBA-zno)	Breast	MCF-7	40 nm	↑ apoptotic frequency, ↑ mitochondrial damage,	5–50 μg/ml	[87]
6	Polymeric (chitosan) nanoparticles	Lung	A549	<200 nm	↑ release, ↑ cytotoxicity to cancer cells	12.5, 25, 50, 75, 100, 150 and 200 μM	[88]
7	Polymeric (chitosan) nanoparticles	Breast	MDA MB 468	<200 nm	↑ release, ↑ cytotoxicity to cancer cells.	12.5, 25, 50, 75, 100, 150 and 200 μM	[88]
8	Polymeric micelles	Ovarian Multidrug resistant Breast	Skov-3 NCI/ADR MCF-7 and MDA-MB-231	24.83 to 45.88 nm	↓ cell viability, ↑ targeted drug release directly into the intracellular environment	6.25 to 100 μM	[89]
9	Gold-quercetin into poly (DL-lactide-co-glycolide) nanoparticles	Cervical	Male BALB/c nu/nu nude mice xeno-grafted with Caski cells	--	↓ tumor xenograft growth and development, ↑ KI-67, ↑ Bax, ↑ Bad, ↑ Cyto-c, ↑ ↓S6RP	--	[90]

**Table 2:** Preclinical studies showing apoptotic effects of quercetin on liver cancer.

Type of Cancer	Cells	Effects	Mechanistic Insight	Concentration	References
Hepatocellular	HepG2	Induces Apoptosis, Cell Cycle arrest	↓ Proliferation of cancer cells, ↑ cell cycle arrest at S and G1 phase, ↑ necrotic and apoptotic cells,	Quercetin 20 to 220 µM or sorafenib 5 to 40 µM)	[78]
Hepatocellular	HepG2	Induces Apoptosis	↓ Cell proliferation, ↓ Bcl-2, ↓ mitochondrial mass, ↓ mitochondrial membrane potential, ↑ mitochondrial superoxide anion, ↑Caspases 3/7/9, ↓↑ BAX,	Quercetin and Permethylated Anigopreissin A (PAA) (inhibitors of hGDH1)-1,2,4,8,12 and 16 μM	[94]
Hepatoblastoma	HepG2, HuH-6 and HepT1	Induces Apoptosis,	↑ SIRT6, upregulation of SIRT6, suppressed cell proliferation and invasion, promoted cell apoptosis, ↓ frizzled 4 (FZD4) transcription, ↓ FZD4 and H3K9ac, ↓ Wnt5a, β-catenin, cyclin D1 and c-Myc	0, 60 and 120 mM	[95]
Hepatocellular	HepG2, Huh-7 (Gemcitabine resistant)	Induces Apoptosis, Cell Cycle arrest	↓ Proliferation of gemcitabine resistant cancer cells, ↑ apoptosis frequency, ↑ gemcitabine efficacy, ↑ accumulation of HepG2 cells in S phase, ↓ G1 and G2/M phase populations, ↑ p53, ↓ cyclin D1	Quercetin 0, 10, 25, 50, 100, or 200 μM or 0, 10, 25, 50, 100, and 200 nM Gemcitabine	[95,96]
Liver	KIM-1, KYN-1, KYN-2, KYN-3, HAK-1A, HAK-1B, HAK-2, HAK-3, HAK-4, HAK-5, and HAK-6	Induces Apoptosis, Cell Cycle arrest	↓ Cell proliferation, ↓ viable cell count, ↑ apoptosis frequency, G0/G1, G2/M and S phase cell cycle arrest	0–100 μM	[77]
Hepatocellular	HepG2 and Huh7	Induces Apoptosis	↓ cell viability and colony growth, ↑ apoptotic pathway, ↑ caspases ↑ Bax	100–500 μM	[97]
Hepatocellular	HepG2	Induces Apoptosis,	↑ cell viability, ↑ caspase-3 and 8, ↑ loss in cell connections, ↑ cell shrinkage, ↑ cell surface detachment, ↑ cytoplasmic density, ↑ dead cells, ↑ Bax, Bid, Bad, and p53, ↓ Bcl-2 and Bcl-XL, ↑ GRP78 and CHOP	Combination of naringenin, quercetin, and naringin and balsamin IC50 values for Nar, Nir and Qu are 150 mM, 20 mM and 37 mM, respectively, and 25 µg/mL of balsamin	[98]
Hepatocellular	SMMC7721 and HepG2	Induces Apoptosis, Cell Cycle arrest	↓ Growth of HCC cells, ↑ autophagosomes and autolysosomes, ↑ LC3A/B-II and beclin1, ↓ p62, ↓ phosphorylated AKT, mTOR, p70S6K and 4EBP1, ↑ phosphorylated JNK, ERK1/2 and p38MAPK	IC50’s at 21.0 and 34.0 μM	[48]
Hepatocellular	LM3	Induces Apoptosis, Cell Cycle arrest	↓ Cancer cell viability, ↑ apoptosis frequency, ↑ cleaved DNA, ↑ cells were arrested in the S and G2/M phases, ↓ G0/G1 phase cells, ↓ vimentin and MMP9, ↓ invasion and migration, ↑ LC3, ↓ p-STAT3	0, 20, 40, 60, 80, 100,120, 140, 160, and 200 µmol/L	[32]
Hepatocellular	HepG2	Induces Apoptosis, Cell Cycle arrest	↓ Proliferation of cancer cells, ↓ intracellular ROS level, ↓cyclin E and SOD1	--	[99]
Hepatocellular	HepG2	Induces Apoptosis	↓ Cell viability, ↑ cell apoptosis, ↓ chymotrypsin-like activity of proteasome, ↑ cleaved caspase-3, ↓ Bcl-2, ↑ p38 MAPK and JNK phosphorylation, ↓ ERK1/2 phosphorylation	0, 25, 50, and 100 µM	[62]
Liver	MHCC97H, Hep3B, HCCLM3 and Bel7402	Induces Apoptosis,	↓ Cancer cell proliferation, cell migration and colony formation, ↑ caspases, ↑ cytochrome c, ↓ NF-κB ↓ Akt and ERK1/2, P-27 was expressed highly, ↓ c-Myc, ↓ cyclin-D1, ↓ CDK1, ↓ MMP7, ↓ β-catenin,	Gold-quercetin -poly(DL-lactideco-glycolide) nanoparticles-0,10,20,30,40,50 and 50 µg/mL	[49]
Hepatocellular	SMMC-7721, HepG2 and HuH-7	Induces Apoptosis	↑ ZD55-TRAIL ↑ caspases and cleaved PARP, ↓ ZD55-TRAIL mediated NF-κB activation, ↑ pro-apoptotic action of ZD55-TRAI, ↓ Bcl-2, ↑ Bax	ZD55-TRAIL adenovirus-1,2,5,10 MOI + Quercetin 5, 10, 25, 50 μM	[79]
Hepatocellular	HepG2 and SMCC-7721	Induces Apoptosis, Cell Cycle arrest	↓ Cell proliferation, ↑ apoptosis, ↑ Bad and Bax, ↓ Bcl-2 and Survivin, ↑ 5-fluorouracil (5-FU) therapeutic efficacy, ↓ cells in S phases, ↑ cells in the G0/G1 phase	0.05, 0.1, and 0.15 mmol/L	[28]
Hepatocellular	HepG2	Apoptosis	↓ Cell growth, ↑ apoptosis, ↑ nuclear condensation and fragmentation, ↓ Sp1 and Sp1 regulatory protein, ↑ p27, p21, ↑ Bax, ↑ caspases and cleaved PARP	10–100 µM	[100]
Liver	HepG2 and Hep3B	Induces Apoptosis, Cell Cycle arrest	↑ Apoptosis, ↑ caspase-3, -8 and -9, ↓ phosphorylation of ERK and p38MAPK, ↑ phosphorylation JNK, ↓ PKC, entering the S and the G2/M phases gradually decreased, while most cells were blocked in the G1 phase	0, 100, 200, 400 and 800 µM	[101]
Hepatocellular	HepG2	Induces Apoptosis	↓ Cell proliferation, blockade of the cell cycle in the S-phase, ↓ DNA topoisomerase II, ↑ DNA fragmentation, ↑ caspase-3, ↑ apoptosis frequency	1, 10, 50, 100, 150 and 200 μM	[102]
Hepatoma	HepG2	Induces Apoptosis	↓Cell viability, ↑ ROS generation, ↑ caspase-3 and -9, ↑↓ caspase-8, ↓ Bcl-xL:Bcl-xS ratio, ↑ translocation of Bax to the mitochondrial membrane, ↓ Akt and ERK	0–100 μM	[30]
Hepatoma	H22, LL/2	Induces Apoptosis	↓ cell proliferation, ↑ apoptotic cell (sub-G1 cells)	0,5,10 and 15 μg	[103]

**Table 3:** Apoptotic effects of quercetin on liver cancer based on in vivo studies.

Type of Cancer	Animal Models	Mechanisms	Dosage	Duration	References
Hepatocellular	Chemically induced HCC rat model with injection of Diethylnitrosamine @200 mg/kg	↓ Liver enzymes—aminotransferase (ALT), ↓ aspartate aminotransferase (AST), ↓ alkaline phosphatase (ALP), ↓ total proteins (TP) and conjugated bilirubin (direct bilirubin), ↓ C-reactive protein (CRP), ↓ interleukin 6 (IL-6), ↓ lactate dehydrogenase (LDH), ↓ PIVKA-II and AFP, ↓ Ki-67 cells, ↓ TNFa, VEGF, p53 and NF-κB expression	Quercetin 50 mg/kg + sorafenib 7.5 mg/kg	13 weeks	[78]
Hepatoblastoma	BALB/c nude mice bearing HepG2 cells (1 × 10^7^) cells	↓ Tumors grew slower and size, ↓ weighed, ↑ SIRT6, ↓ FZD4	10 mg/kg	28 days	[78]
Hepatocellular	BALB/c nude mice bearing SMMC7721 cells (2 × 10^6^) cells	↑ Autophagosomes and autolysosomes, ↓ AKT/mTOR, ↑ MAPK, ↑cleaved caspase-3, ↑ BAX, ↓ Bcl-2, ↑ LC3A/B, ↓ p62, ↑ necrosis	60 mg/kg	10 days	[48]
Hepatocellular	Sprague Dawley rats (TAA Induced)	↓ Caspase-3, ↓↑ caspase-8, ↑ p53, ↓ cyclin D1 and Ki-67	TAA -200 mg/kg + Quercetin 100 mg/kg	21 days	[29]
Hepatocellular	Nude mice bearing LM3 cells	↓ Tumor volume, ↑ necrosis, ↑ TUNEL-positive cells, ↓ PCNA, ↑ Bax	100 mg/kg	21 days	[32]
Hepatocellular	BALB/c nu/nu nude mice bearing MHCC97H cells (1 × 10^7^ cells)	↓ Tumor volumes, ↓ AP-2β and COX2 levels, ↑ TUNEL levels, ↓ cleaved caspase-9, ↓ cleaved caspase-3, ↓ cytoplasm Cyto-c, ↓ phosphorylated IKKα, IκBα and NF-κB	30, 40 and 50 mg/kg Quercetin nanoparticles	35 days	[49]
Hepatocellular	BALB/C nude mice bearing HuH-cells	↓ Tumor volume, ↑ survival rate	ZD55-TRAIL was injected intra-tumorally at 1 × 10^9^ plaque-forming units + Quercetin 150 mg/kg	49 days	[79]
Hepatocellular	BALB/c nude mice	↑ 5-fluorouracil (5-FU) therapeutic efficacy, ↑ apoptosis, ↓ tumor growth	Quercetin 40 mg/kg and 5-fluorouracil 30 mg/kg	23 days	[28]
Liver	Wistar rats (two-phase model of hepato carcinogenesis)	↓ Number and volume of preneoplastic lesions, ↑ apoptosis, ↓ proliferative index, ↓ cell percentages ↓ M phase, ↓ cyclin D1, ↓ cyclin A, ↓ cyclin B, ↓ cyclin-dependent kinase 1, ↑ peroxisome proliferator, ↑ caspase-3 activity, ↑ Bax/Bcl-2, ↑ cytosolic cytochrome c	10 and 20 mg/kg	42 days	[27]
Hepatoma	C57BL/6N mice bearing LL/2 Lewis lung cancer (1 × 10^6^)	↓ Tumor growth, ↓ tumor volume, ↓ HSP70, ↑ apoptosis rate	Liposomal Quercetin -50 mg/kg	15 days	[103]
Hepatoma	BALB/c mice bearing H22 tumor models (5 × 10^5^)	↓ Tumor growth, ↓ tumor volume, ↓ HSP70, ↑ apoptosis rate	Liposomal Quercetin -50 mg/kg	15 days	[103]
